# Circulating adrenal and gonadal steroid hormones heterogeneity in active young males and the contribution of 11-oxy androgens

**DOI:** 10.1038/s41598-024-66749-9

**Published:** 2024-07-14

**Authors:** Amanda C. Swart, Desmaré van Rooyen, Therina du Toit, Bianca Heyns, John Molphy, Mathew Wilson, Roisin Leahy, Stephen L. Atkin

**Affiliations:** 1 Department of Biochemistry, Stellenbosch University, Stellenbosch, 7600 South Africa; 2 Department of Chemistry and Polymer Science, Stellenbosch University, Stellenbosch, 7600 South Africa; 3 Research Institute of Sport and Exercise Sciences, Liverpool John Moores University, Liverpool, UK; 4 Institute of Sport, Exercise and Health, University College London, London, WC1E 6BT UK; 5 Data Science Centre, School of Population Health, Royal College of Surgeons in Ireland, University of Medicine and Health Sciences, Dublin 2, Ireland; 6 Royal College of Surgeons in Ireland, Busaiteen, Bahrain; 7 Weill Cornell Medicine Qatar, Doha, Qatar

**Keywords:** Androgen replacement therapy, Hypogonadism, 11β-hydroxyandrostenedione (11OHA4 11OHAD11OHAn), 11-ketotestosterone (11KT), Dehydroepiandrosterone (DHEA), Cytochrome P450 11β hydroxylase (CYP11B1), Biochemistry, Endocrinology

## Abstract

The classical androgens, testosterone and dihydrotestosterone, together with dehydroepiandrosterone, the precusrsor to all androgens, are generally included in diagnostic steroid evaluations of androgen excess and deficiency disorders and monitored in androgen replacement and androgen suppressive therapies. The C11-oxy androgens also contribute to androgen excess disorders and are still often excluded from clinical and research-based steroids analysis. The contribution of the C11-oxy androgens to the androgen pool has not been considered in androgen deficiency. An exploratory investigation into circulating adrenal and gonadal steroid hormones in men was undertaken as neither the classical androgens nor the C11-oxy androgens have been evaluated in the context of concurrent measurement of all adrenal steroid hormones. Serum androgens, mineralocorticoids, glucocorticoids, progesterones and androgens were assessed in 70 healthy young men using ultra high performance supercritical fluid chromatography and tandem mass spectrometry. Testosterone, 24.5 nmol/L was the most prominent androgen detected in all participants while dihydrotestosterone, 1.23 nmol/L, was only detected in 25% of the participants. The 11-oxy androgens were present in most of the participants with 11-hydroxyandrostenedione, 3.37 nmol, in 98.5%, 11-ketoandrostenedione 0.764 in 77%, 11-hydroxytestosterone, 0.567 in 96% and 11-ketotestosterone: 0.440 in 63%. A third of the participants with normal testosterone and comparable 11-ketotestosterone, had significantly lower dehydroepiandrosterone (*p* < 0.001). In these males 11-hydroxyandrostenedione (*p* < 0.001), 11-ketoandrostenedione (*p* < 0.01) and 11-hydroxytestosterone (*p* < 0.006) were decreased. Glucocorticoids were also lower: cortisol (*p* < 0.001), corticosterone (*p* < 0.001), cortisone (*p* < 0.006) 11-dehydrocorticosterone (*p* < 0.001) as well as cortisol:cortisone (*p* < 0.001). The presence of dehydroepiandrosterone was associated with 16-hydroxyprogesterone (*p* < 0.001), which was also significantly lower. Adrenal and gonadal steroid analysis showed unexpected steroid heterogeneity in normal young men. Testosterone constitutes 78% of the circulating free androgens with the 11-oxy androgens abundantly present in all participants significantly contributing 22%. In addition, a subset of men were identified with low circulating dehydroepiandrosterone who showed altered adrenal steroids with decreased glucocorticoids and decreased C11-oxy androgens. Analysis of the classical and 11-oxy androgens with the additional measurement of dehydroepiandrosterone and 16-hydroxyprogesterone may allow better diagnostic accuracy in androgen excess or deficiency.

## Introduction

Diagnostic quantitative androgen analysis is essential to investigate androgen excess disorders, primary or secondary hypogonadism, and to determine androgen levels in replacement or suppressive therapies in men. Although total testosterone (T) is clinically measured for androgen sufficiency, only the free form of T is bioactive which is estimated indirectly using the free androgen index. However, bioactive androgens are not limited to T alone. Active functional androgens bind and activate the androgen receptor (AR) and regulate androgen-responsive genes which are critical in the development and maintenance of male sexual characteristics. Steroid metabolism is complex, yielding potent functional steroids that are not all measured in clinical practice.

**Figure 1:**
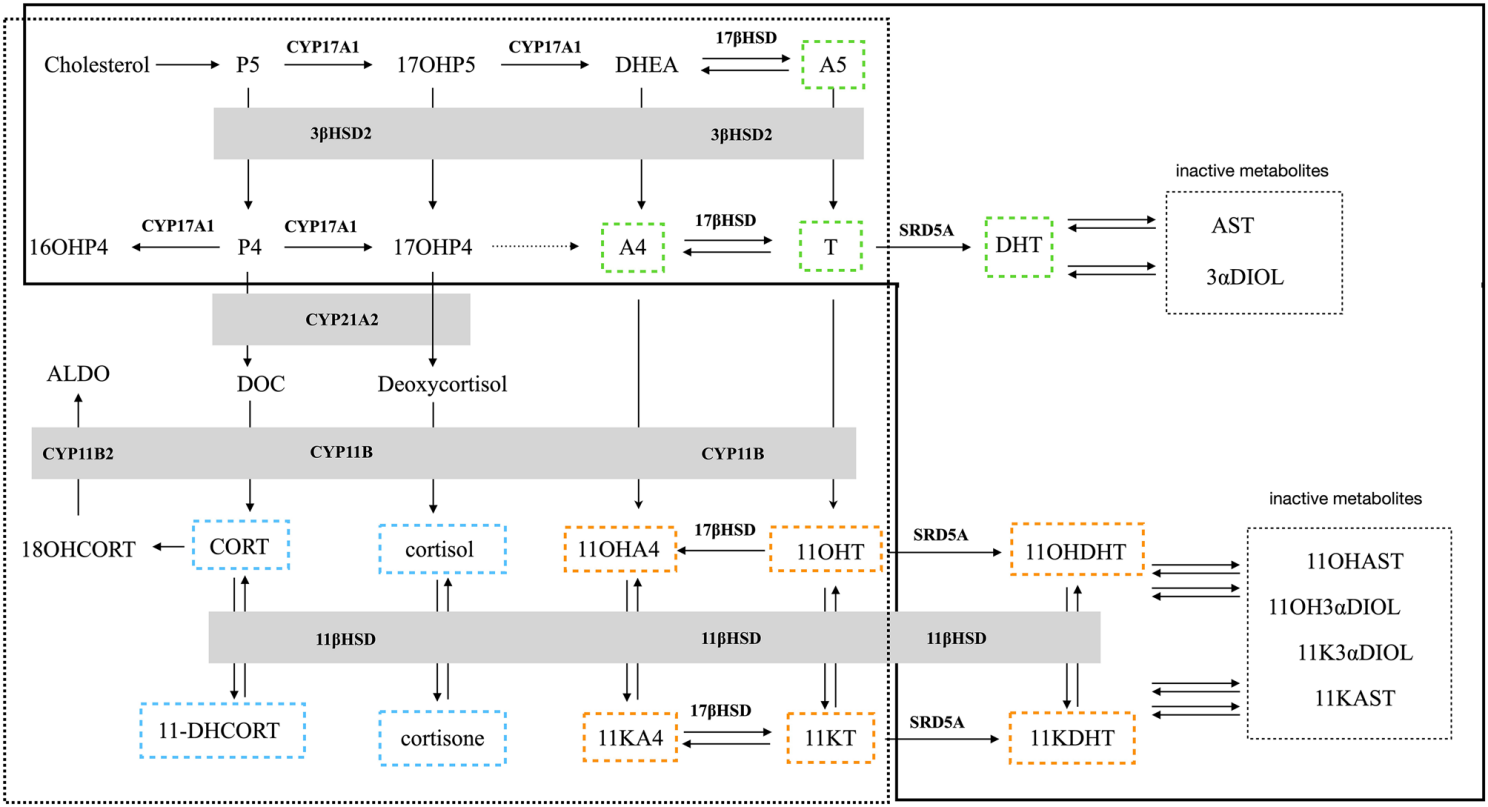
Steroid biosynthesis in the adrenal, gonads and peripheral tissue. Adrenal production, boxed, black dotted lines and gonadal production, boxed, black solid lines. Reactions are catalysed by CYP17A1, cytochrome P450 17α-hydroxylase; CYP21A2, cytochrome P450 21-hydroxylase; CYP11B1, cytochrome P450 11β-hydroxylase; CYP11B2, aldosterone synthase; 3βHSD2, 3β-hydroxysteroid dehydrogenase type 2; 11βHSD, 11β-hydroxysteroid dehydrogenase; 17βHSD, 17β-hydroxysteroid dehydrogenase and SRD5A, steroid 5α-reductase. Steroids include the androgen precursor: DHEA, dehydroepiandrosterone; (i) classical androgens: A5, androstenediol; A4, androstenedione; DHT, dihydrotestosterone; T, testosterone; AST, androsterone; 3αAdiol, 5α-androstane-3α,17β-diol; (ii) C11-oxy androgens: 11OHA4, 11β-hydroxyandrostenedione; 11KA4, 11 ketoandrostenedione; 11OHT, 11β-hydroxytestosterone; 11KT, 11-ketotestosterone; 11OHDHT, 11β-hydroxydihydrotestosterone; 11KDHT, 11-ketodihydrotestosterone; 11OHAST, 11β-hydroxyandrosterone; 11KAST, 11-ketoandrosterone; 11OH3αDIOL, 5α-androstan-3α,11β,17β-triol; 11K3αDIOL, 5α-androstan-3α,17β-diol-11-one. (iii) progesterones: P5, pregnenolone; 17OHP5, 17α-hydroxypregnenolone; P4, progesterone; 17OHP4, 17α-hydroxyprogesterone; 16OHP4, 16α-hydroxyprogesterone; (iv) mineralo- and glucocorticoids: DOC, 11-deoxycorticosterone; CORT, corticosterone; 18OHCORT, 18-hydroxycorticosterone; ALDO, aldosterone; 11-DHCORT, 11-dehydrocorticosterone. Blue boxed steroids, glucocorticoids; green boxed steroids, classical androgens; orange boxed steroids, C11-oxy androgens.

Dehydroepiandrosterone (DHEA) is the precursor to all androgens that can be divided into two classes: the classical androgens and the C11-oxy androgens. The C11-oxy androgens differ from the classical androgens only in that they have either a hydroxy or oxo group at carbon 11 (the latter also generally referred to as a keto group). The classical androgens include androstenedione (A4), T and dihydrotestosterone (DHT) and their inactive metabolites. The C11-oxy androgens include 11-hydroxyandrostenedione (11OHA4), 11-hydroxytestosterone (11OHT), 11-ketoandrostenedione (11KA4), 11-ketotestosterone (11KT), 11-hydroxydihydrotestosterone (11OHDHT), 11-ketodihydrotestosterone (11KDHT) and their inactive metabolites^1^. The classical androgens are produced primarily in the gonads and in the adrenal albeit to a lesser degree and with the exception of dihydrotestosterone (DHT) and the respective metabolites as shown in Fig. 1. It was generally accepted for decades, that the only androgens of importance were the classical androgens, T and DHT. In women, it was believed that testosterone produced in the adrenal was the only androgen source. The C11-oxy androgens produced in the adrenal were not considered in the context of normal androgens in either gender. Since the C11-oxy androgens include active androgens, 11KT, 11KDHT, and 11OHDHT^2^, these steroids add to the overall androgenicity of the androgen steroid pool in both males and females, supplementing the action of the classical active androgens.

The metabolic pathways originating from DHEA therefore form classical androgens and C11-oxy androgens yeilding potent downstream androgens. In these pathways, the classical androgens and the C11-oxy androgens contribute active functional androgens, T, DHT, 11KT, 11OHDHT and 11KDHT to the endogenous androgen pool (Fig. 1). The C11-oxy progesterones which may potentially also form potent downstream androgens, and the C11-oxy androgens are only produced in the adrenal gland by cytochrome P450 11β-hydroxylase (CYP11B1), an enzyme generally known to catalyse the production of cortisol. We have shown that CYP11B1 as well as aldosterone synthase (CYP11B2) which catalyses the production of aldosterone (ALDO), also catalyses the biosynthesis of 11OHA4, 11OHT, 21-deoxycortisol (21dF) and 11β-hydroxyprogesterone (11OHP4). These steroids are subsequently metabolised by 11β-hydroxysteroid dehydrogenase by converting the hydroxyl group at carbon 11 to an oxo (keto) group in the adrenal and peripheral tissue^3–5^. 11βHSD2 has long only been associated with the conversion or inactivation of cortisol to cortisone. The conversion of 11OHA4 and 11OHT by 11βHSD2 yields 11KA4 and 11KT, respectively, with the subsequent production of 11OHDHT from 11OHT and 11KDHT from 11KT, resulting in potent active androgens capable of binding and acivating the AR.

Studies using validated methods report circulating 11OHA4 and 11KT are 2- to 3- fold higher in young males (12–18 years) compared to younger boys^6^ and all four circulating C11-oxy androgen concentrations are reported in ages 21 to 95 years. 11KT concentrations were comparable between studies, though 11OHA4, and 11KA4 concentrations varied between twofold and tenfold, respectively^7,8^; only one study having included 11KDHT and downstream metabolites, 11β-hydroxyandrosterone (11OHAST) and 11-ketoandrosterone (11KAST)^7^. Another study reported circulating 11OHA4, 11KA4 and 11KT levels in men 19–59 years of age, though 11OHT was undetectable^9^.

Androgen excess conditions show high C11-oxy androgens, such as classic congenital adrenal hyperplasia (CAH); 21-hydroxylase deficiency (21OHD); polycystic ovary syndrome (PCOS); prostate cancer (PCa); benign prostate hyperplasia (BPH) and premature adrenarche; however, due technical measurement difficulties, 11OHA4, 11OHT, 11KA4, 11KT, 11OHP4 and 21dF evaluations are scarce. Their specific and accurate measurement is important as 11KT is the predominant active androgen in castration-resistant prostate cancer (CRPC)^10^ with both 11OHA4 and 11KDHT detected in PCa tissue, tissue homogenates, together with 11KA4, 11KT and 11OHT in serum and plasma^11–13^. Moreover, in BPH patients, 11OHP4, 11-ketoprogesterone (11KP4) and 11-ketodihydroprogesterone (11K-DHP4) are found in the circulation and in prostate tissue^12^. These data suggest that the C11-oxy androgens contribute to 11KDHT levels in vivo. In addition, it is possible that T replacement therapy and anti-androgen therapy may affect the regulation of the C11-oxy androgens, potentially modulating AR interactions and downstream consequences.

The technical difficulties measuring the classical and C11-oxy androgens in the context of the adrenal hormone pool have limited their clinical evaluation and assessments; therefore, this study was to undertake a comprehensive, exploratory analysis of all circulating adrenal and gonadal steroids in healthy male subjects, determining the contribution of the C11-oxy androgens to the overall steroid profile using state of the art ultrahigh performance supercritical fluid chromatography and tandem mass spectrometry (UHPSFC-MS/MS).

## Results

### Demographic characteristics of participants

There were 71 participants with a mean age of 25.1 years. Participants were mainly of Arab (54%) or Black (27%) ethnicity. Descriptive statistics of all participants are presented in Table 1. Participant demographics showed participants did not differ significantly on any demographic characteristics (p > 0.05).

**Table 1:** Demographic characteristics all participants (n = 71).

	Overall (N = 71)
Age	
Mean (SD)	25.1 (4.77)
Median (Q1, Q3)	24.0 (21.0, 28.0)
Ethnicity	
Arab	38 (53.5%)
Black	19 (26.8%)
White	12 (16.9%)
Asian	2 (2.8%)
Exercise	
Mean (SD)	13.4 (5.61)
Median (Q1, Q3)	12.0 (11.3, 14.0)
Height (cm)	
Mean (SD)	181 (11.5)
Median (Q1, Q3)	181 (172, 188)
Body mass (kg)	
Mean (SD)	78.6 (15.2)
Median (Q1, Q3)	76.0 (65.4, 88.8)
BMI	
Mean (SD)	23.8 (2.72)
Median (Q1, Q3)	23.7 (21.7, 25.3)
BMI category	
Underweight/normal	48 (67.6%)
Overweight/obese	21 (29.6%)
BSA	
Mean (SD)	1.99 (0.243)
Median (Q1, Q3)	1.93 (1.79, 2.17)

### UHPSFC-MS/MS analysis of free unconjugated adrenal and gonadal steroid hormones

Circulating mineralocorticoids, glucocorticoids and androgens (Supple. file, Supple. Table 1) and many steroids, being below the lower limit of detection (LOD) (Supple. Table 2) were therefore not detected. The classical androgens that were not detected were 5αDIONE and 3αDIOL and the C11-oxy androgens not detected were 11OH-5αDIONE, 11K-5αDIONE, 11OHDHT, 11KDHT, 11OHAST, 11KAST and 11OH3αADIOL. The C11-oxy progesterones that were not detected included 21dF, 21dE, Pdione, 11αOHP4, 11αOH-DHP4, 11βOHP4, 11KP4, 11βOHDHP4, 11KDHP4, 11OHPdiol, alfaxalone, 11OH-Pdione, 11K-Pdione, and 11K-Pdiol. DOC was the only adrenal steroid not detected at all while others were detected but at low frequencies: 11-deoxycortisol (0.37—2.15 nmol/L, 6/70), 18OHCORT (1.64–32.96 nmol/L, 4/70), ALDO (1.0—15.15 nmol/L, 3/70). Two C11-oxy steroids were only detected in three participants, 11K3αADIOL (1.28, 2.76 nmol/L, 2/70) and 3,11diOH-DHP4 (3.4 nmol/L, 1/70), and therefore not included in further analysis. Progesterones detected and not included in analysis were DHP4 (6.58 nmol/L, 1/70). Pdiol (7.36 nmol/L, 1/70) and pregnanetriol (22.26 nmol/L, 1/70).

Steroids subsequently included in analyses are listed in Table 2, showing the percentage at which each steroids was detected above lower limit of quantification (LLOQ) enabling accurate quantification and below the LLOQ but above the LOD. Steroids detected below the LOD in more than 75% of participant were not included in analyses.

**Table 2:** Representation of steroid detected in 70 heathy young males at frequencies above 25%.

Steroid	Above LLOQ (%)	Below LLOQ (%)	Below LOD (%)
Testosterone (T)	100.00	0.00	0.00
Cortisol	100.00	0.00	0.00
Cortisone	100.00	0.00	0.00
Corticosterone (CORT)	82.86	17.14	0.00
11-Dehydrocorticosterone (11-DHCORT)	78.57	21.43	0.00
16α hydroxyprogesterone (16OHP4)	77.14	21.43	1.43
11α-hydroxytestosterone (11OHT)	70.00	25.71	4.29
11α-hydroxyandrostenedione (11OHA4)	51.43	47.14	1.43
Dihydrotestosterone (DHT)	15.71	11.43	72.86
Progesterone (P4)	12.86	12.86	74.29
Androstenedione (A4)	8.57	74.29	17.14
17α hydroxyprogesterone (17OHP4)	2.86	40.00	57.14
Dehydroepiandrosterone (DHEA)	1.43	84.29	14.29
11-ketoandrostenedione (11KA4)	0.00	77.14	22.86
Androstenediol (A5)	0.00	71.43	28.57
11-ketotestosterone (11KT)	0.00	62.86	37.14
17α-hydroxypregnenolone (17OHP5)	0.00	62.86	37.14
Pregnenolone (P5)	0.00	28.57	71.43

Percentage (%) represents the number of participants in which each steroid was detected above the lower limit of detection (LLOQ), below the LLOQ but above the limit of detection (LOD) and undetected below the LOD.

Adrenal and gonadal steroids present at frequencies higher than 25% were there analysed and included mineralocorticoids, glucocorticoids and androgens. The steroid profile of one participant differed markedly from other profiles in the cohort. All the steroids analysed were below the LOD except for T, possibly indicative of an underlying clinical condition and was subsequently was excluded from analysis.

### Analysis of steroids present at frequencies higher than 25% in participants

Steroid analysis of the participant group (n = 70) showed that while T, 24.5 nmol/L, was detected in all participants falling within the normal range, the hormone spanned a wide range 7.76–47.3 nmol/L. T precursors, A5 and A4, which were detected in 72% and 83% of participants, respectively, showed A4 concentrations 2.7-fold lower than A5. DHT, the potent SRD5A metabolite of T, was detected in far fewer participants, only 27% participants had detectable concentrations. No classical inactive androgens were detected (Fig. 1). Although the classical androgen levels were higher than the C11-oxy androgen levels, the latter were detected in a more participants with 11OHA4, 11OHT, 11KA4 and 11KT detected in 98.6, 95.7, 77 and 63% of the participants, respectively. 11OHA4 was detected at highest concentration followed by 11KA4 with 11OHT and 11KT which were all lower than 1 nmol/L. Taken together the C11-oxy androgens were present at far lower levels than the classical androgens.

Although the C11-oxy androgens were detected, no C11-oxy progesterones were detected. All of the progesterones were present at far lower concentrations than androgens with pregnenolone (P5) and progesterone (P4) detected in ~ 25% of participants. Of interest was 16α-hydroxyprogesterone (16OHP4), the product of P4, which was detected in 98.6% of participants, the most abundant progesterone detected.

Also detected in all participants were the glucocorticoids, cortisol and corticosterone (CORT) and their respective inactive forms, cortisone and 11-dehydrocorticosterone (11-DHCORT). Cortisol and cortisone were the most abundant steroids at 338 and 55.8 nmol/L, respectively with means all within the normal range. Deoxycortisol, the precursor steroid of cortisol was detected at very low levels in only 8% of participants. The inactive metabolites of cortisol and CORT, catalysed by 11βHSD2, cortisone and 11-DHCORT, were detected at lower levels than their active precursors, respectively. In addition, CORT levels as well as cortisol:cortisone were all within normal ranges. The downstream metabolite in the mineralocorticoid pathway, 18-hydroxycorticosterone (18OH-CORT) was detected in only four participants and aldosterone (ALDO) in three participants.

Upon further inspection it was evident that some participants had very low cortisol and cortisone concentrations together with low cortisol:cortisone. Cluster analysis showed that DHEA in these participants were within the lower normal range of circulating DHEA. We therefore analysed adrenal and gonadal steroids dividing the participants into two groups: those in the lower tertile (6.9 nmol/L), comprising 34% of the participants, and those with DHEA concentrations above (66%). Participant demographics of the participants with DHEA below and DHEA above 7 nmol/L showed participants in the two groups did not differ significantly on any demographic characteristics (p > 0.05) (Supple. Table 3.)

Comparing participants with low circulating DHEA (DHEA < 7 nmol/L) to those with DHEA > 7 nmol/L (Table 3), showed that although steroid profiles differed considerably, the classical androgen (A5, A4, T, DHT) levels remained comparable between the two groups. In contrast, the C11-oxyandrogens with the exception of 11KT were significantly lower in the group with low DHEA. In addition, together with significantly lower DHEA, the progesterones detected, P5, 17OHP5 and 16OHP4 were also significantly lower. The active glucocorticoids, cortisol and CORT, their inactive forms, cortisone and 11-DHCORT as well as cortisol:cortisone were also significantly lower in the group with low DHEA. Further analysis also showed that the presence of DHEA was significantly associated with 16OHP4 (p < 0.001) (Suppl. Table 4). The presence of DHEA was not significantly associated with any other steroid (p > 0.05).

**Table 3:** Adrenal and gonadal steroid concentrations of healthy males, 18 -35 years of age.

STEROID nmol/L	Overall (n = 70)	DHEA > 7 nmol/L (n = 47)	DHEA < 7 nmol/L (n = 23)	P-value
Dehydroepiandrosterone (DHEA)_				
Mean (SD)	12.1 (9.07)	16.1 (8.58)	4.04 (1.44)	< 0.001
Median (Q1, Q3)	9.64 (5.19, 18.1)	13.5 (9.64, 20.6)	3.45 (3.45, 5.09)	
Min–Max	0.487–52.7	6.93–52.7	0.487–6.64	
Androstenediol (A5)				
Mean (SD)	4.40 (2.44)	4.79 (2.79)	3.59 (1.13)	0.107
Median (Q1, Q3)	3.40 (3.40, 4.88)	3.77 (3.40, 5.98)	3.40 (3.40, 3.87)	
Min–Max	0.479–13.4	0.479–13.4	1.24–6.68	
Androstenedione (A4)				
Mean (SD)	1.63 (1.65)	1.71 (1.86)	1.44 (1.07)	0.713
Median (Q1, Q3)	1.08 (0.350, 2.47)	0.942 (0.350, 2.51)	1.15 (0.537, 2.17)	
Min–Max	0.0651–7.53	0.0651–7.53	0.168–4.25	
Testosterone (T)				
Mean (SD)	24.5 (6.85)	25.5 (6.60)	22.6 (7.08)	0.148
Median (Q1, Q3)	24.3 (20.3, 28.9)	25.3 (21.3, 29.5)	22.8 (18.5, 28.0)	
Min–Max	7.76–47.3	7.76–47.3	7.81–35.4	
Dihydrotestosterone (DHT)				
Mean (SD)	1.23 (2.90)	1.30 (3.01)	1.07 (2.73)	1
Median (Q1, Q3)	0.170 (0.170, 0.170)	0.170 (0.170, 0.170)	0.170 (0.170, 0.334)	
Min–Max	0.0506–15.2	0.0506–15.2	0.114–11.4	
11α-hydroxyandrostenedione (11OHA4)				
Mean (SD)	3.37 (1.75)	4.04 (1.62)	2.00 (1.13)	< 0.001
Median (Q1, Q3)	3.39 (2.09, 4.14)	3.80 (3.17, 4.61)	1.95 (1.12, 2.63)	
Min–Max	0.330–10.4	1.87–10.4	0.330–4.15	
11-ketoandrostenedione (11KA4)				
Mean (SD)	0.764 (0.365)	0.838 (0.361)	0.611 (0.332)	0.010
Median (Q1, Q3)	0.747 (0.382, 1.01)	0.811 (0.607, 1.05)	0.552 (0.330, 0.777)	
Min–Max	0.300–1.79	0.330–1.79	0.300–1.27	
11α-hydroxytestosterone (11OHT)				
Mean (SD)	0.567 (0.518)	0.665 (0.590)	0.367 (0.225)	0.006
Median (Q1, Q3)	0.481 (0.285, 0.670)	0.524 (0.366, 0.738)	0.360 (0.212, 0.505)	
Min–Max	0.0300–3.21	0.0879–3.21	0.0300–0.891	
11-ketotestosterone (11KT)				
Mean (SD)	0.440 (0.328)	0.446 (0.318)	0.427 (0.354)	0.748
Median (Q1, Q3)	0.330 (0.303, 0.464)	0.330 (0.306, 0.530)	0.330 (0.299, 0.419)	
Min–Max	0.0127–1.57	0.0127–1.57	0.0258–1.50	
Pregnenolone (P5)				
Mean (SD)	3.57 (0.978)	3.76 (1.12)	3.18 (0.395)	0.007
Median (Q1, Q3)	3.10 (3.10, 3.63)	3.10 (3.10, 4.14)	3.10 (3.10, 3.10)	
Min–Max	2.72–7.63	2.72–7.63	3.10–4.99	
17α-hydroxypregnenolone (17OHP5)				
Mean (SD)	6.94 (5.86)	8.80 (6.34)	3.13 (1.10)	< 0.001
Median (Q1, Q3)	4.52 (3.00, 8.12)	7.37 (3.45, 11.9)	3.00 (3.00, 3.00)	
Min–Max	0.139–29.3	0.139–29.3	1.03–7.53	
Progesterone (P4)				
Mean (SD)	0.101 (0.209)	0.128 (0.243)	0.0464 (0.0932)	0.406
Median (Q1, Q3)	0 (0, 0.0707)	0 (0, 0.127)	0 (0, 0)	
Min–Max	0–0.839	0–0.839	0–0.271	
17α-hydroxyprogesterone (17OHP4)				
Mean (SD)	0.644 (0.771)	0.511 (0.473)	0.916 (1.13)	0.308
Median (Q1, Q3)	0.300 (0.300, 0.627)	0.300 (0.300, 0.565)	0.300 (0.300, 1.28)	
Min–Max	0.101–4.74	0.133–2.37	0.101–4.74	
16α hydroxyprogesterone (16OHP4)_				
Mean (SD)	0.591 (0.424)	0.725 (0.430)	0.319 (0.248)	< 0.001
Median (Q1, Q3)	0.520 (0.316, 0.763)	0.628 (0.452, 0.828)	0.245 (0.136, 0.432)	
Min–Max	0–2.30	0.183–2.30	0–0.923	
Cortisol_				
Mean (SD)	338 (150)	396 (119)	218 (137)	< 0.001
Median (Q1, Q3)	339 (224, 465)	359 (311, 488)	183 (117, 254)	
Min–Max	58.8–634	192–634	58.8–591	
Corticosterone (CORT)				
Mean (SD)	14.1 (12.1)	18.2 (11.6)	5.73 (8.18)	< 0.001
Median (Q1, Q3)	7.83 (4.93, 22.7)	16.1 (6.98, 27.1)	2.55 (1.50, 6.73)	
Min–Max	0.530–42.9	3.83–42.9	0.530–39.0	
Cortisone_				
Mean (SD)	55.8 (17.4)	59.6 (12.4)	47.8 (23.0)	0.006
Median (Q1, Q3)	56.5 (45.4, 66.1)	60.6 (50.9, 66.5)	47.9 (31.4, 62.7)	
Min–Max	17.8–108	35.1–85.1	17.8–108	
11-dehydrocorticosterone (11-DHCORT)_				
Mean (SD)	4.40 (2.31)	5.26 (2.01)	2.65 (1.88)	< 0.001
Median (Q1, Q3)	4.48 (2.45, 5.82)	5.14 (3.96, 6.71)	2.04 (1.45, 3.00)	
Min–Max	0.470–10.6	1.43–10.6	0.470–7.77	
Cortisol:cortisone				
Mean (SD)	6.20 (2.97)	6.98 (2.86)	4.62 (2.59)	< 0.001
Median (Q1, Q3)	5.33 (4.06, 7.73)	6.59 (4.79, 8.58)	3.63 (3.27, 5.10)	
Min–Max	2.68–17.3	3.46–17.3	2.68–14.9	

Concentrations are shown as the mean with the standard deviation (SD), the median and quartiles together with the minimum and maximum ranges. Data show steroid concentrations of the total participant group (n = 70). Comparison of steroid concentrations of participants with DHEA levels lower than 7 nmol/L (n = 23) and participants with DHEA levels higher than 7 nmol/L (n = 47). P-value indicates significant differences between the two groups.

**Figure 2:**
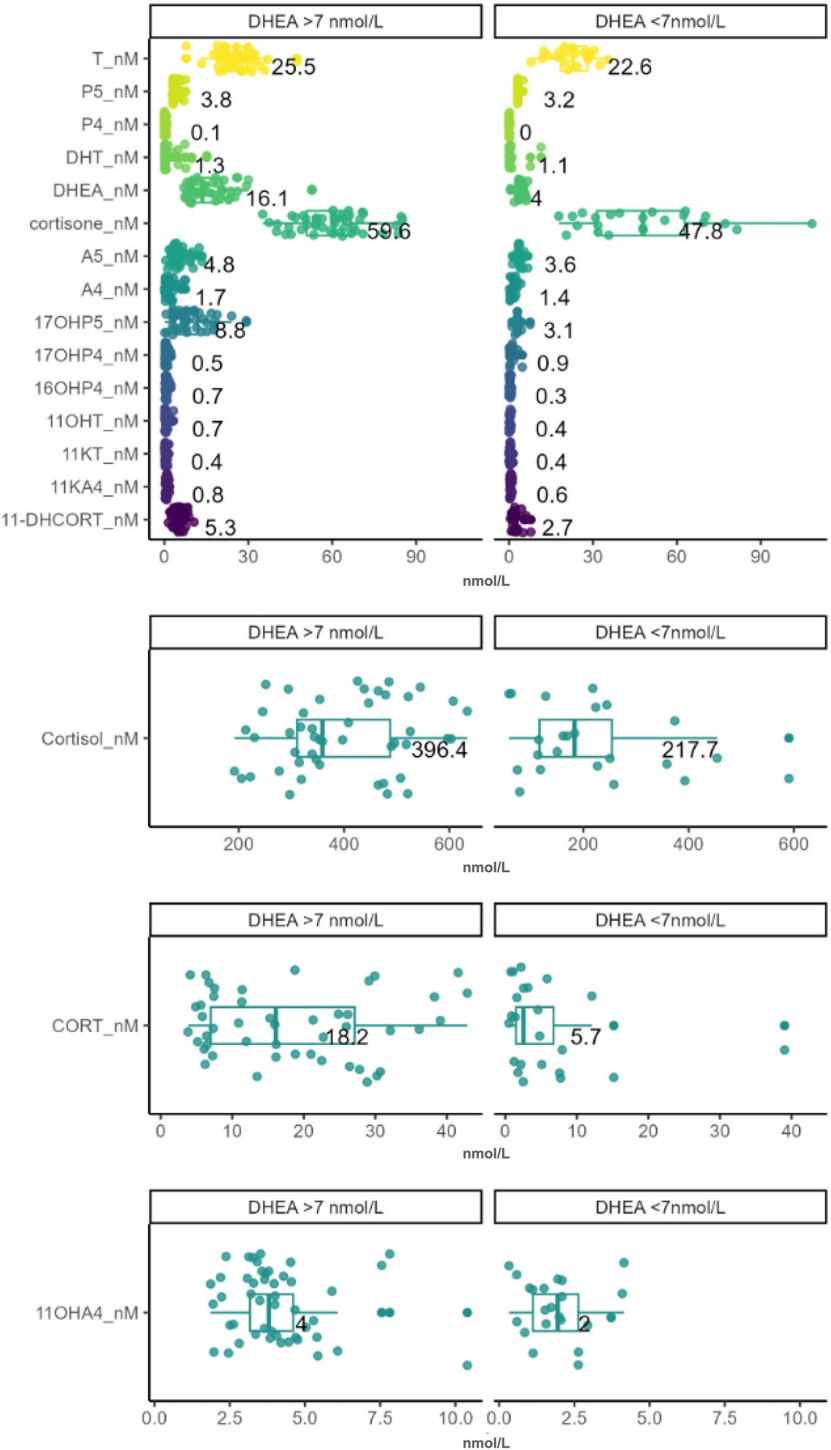
Comparison of steroid levels in participants with DHEA levels higher and lower than 7 nmol/L. Data show mean (SD) of all the measured steroid metabolites.

Considering the mean steroid values of subjects with a DHEA lower than 7 nmol/L, the data clearly showed that the steroid profiles of these males differed markedly when compared to males with higher DHEA levels. Mean steroid concentrations of all steroids measured in this study were lower in males with DHEA lower than 7 nmol/L (Fig. 2), with data showing overall lower adrenal steroids in designated pathways.

## Discussion

This novel study is the first analysis of circulating adrenal and gonadal steroids that included mineralocorticoids, glucocorticoids, progestins and androgens, providing a circulating free-steroid profile in young men. Testosterone was the dominant free circulating classical androgen followed by A4 and DHEA with the levels in accord with the literature with the average mean concentration and range, 1.5–6.0 nmol/L for A4 and 15–40 nmol/L for DHEA between the ages of 18–40 years^14,15^. Interestingly, DHT was not detected in all participants, and the inactive metabolites of the classical androgens, 3αAdiol and AST were not present at all. This may be due to 3αAdiol and AST circulating in their conjugated forms (99% glucuronidated and/or sulphated)^16^ which was not measured in our investigation. Identification and quantification of steroids present at low concentrations would be enhanced by measuring both conjugated as well as free, unconjugated steroids, with only the latter measured in our study. Certain steroids that ionise poorly therefore require to be present at higher concentrations for detection and accurate quantification. The chromatographic separation of numerous steroids, whilst also ensuring the elimination of crosstalk or interference between steroids is challenging. Unique molecular masses of ionised steroids were chosen to identify and quantify (qualifiers and quantifiers) steroids thereby allowing the identification and most accurate quantification of all gonadal and adrenal steroids in a single step. Our findings suggest that the measurement of specific steroid combinations, some of which are not currently included or considered in clinical evaluations of conditions or diseases, could improve the accuracy of diagnosing androgen related conditions^17^. It may also lead to better treatment regimens for T replacement or suppression therapies^18,19^. Androgen deficiency resulting from the hypothalamic-pituitary–gonadal axis dysfunction has received far more attention in men than in women with many features common to both genders—bone density reduction, altered mood states, depression and increased anxiety, impaired cognition, hot flushes, sexual symptoms, decreased muscle mass and increased body fat. Androgen deficiency may not be easily recognised in women as many symptoms are characteristic of the menopause. Since T as well as DHEA levels fall with ageing^15,20,21^, the contribution of the C11-oxy androgens needs to be determined to see if these steroids have an increased androgen contribution in both genders. It is possible that both 11OHA4 and 11OHT produced in the adrenal become a more important androgen source with age, supplementing the classical androgens. The C11-oxy androgens have, to date not been investigated in androgen deficiency.

We did not detect 11KDHT, 11OHDHT or any C11-oxy progesterones in this study and only low levels of DHT, 11OHT and 11KT. This finding suggests that they are unlikely to contribute to androgen activity in health as they are perhaps insufficient for the activation of the AR at a level equivalent to T ^2^. However, the data should be considered with the caveat that circulatory steroid hormones do not necessarily correlate with or reflect steroid levels in tissue, which are likely very different. The presence of the 11-oxy steroids and the potential conversion of C11-oxy progesterones to C11-oxy androgens^3,4^ are in accord with other studies previously investigating hyperandrogenism in which the administration of radiolabelled 21dF resulted in 11OHAST and 11KAST as well as 11K-Pdiol being detected in both a CAH patient and a control subject, while only 11OH- and 11-keto-etiocholanolone (C11-oxy androgen metabolites) were detected in the CAH patient^22,23^. In our study the steroids with the highest detection frequency, after T, were the 11-oxy androgens suggesting age defined reference levels need to be determined for the 11-oxy androgens.

What was surprising was the number of normal healthy participants (33%) that had DHEA levels in the lower normal range (below 7 nmol/L); these males were slightly older, 27.0 (Q1 22.8, Q3 29.5) than those with higher DHEA. While it is generally accepted that DHEA declines with age^24^, this is an unlikely explanation for this relatively young age group. The 11-oxy androgens, 11OHA4, 11KA4 and 11OHT together with 16OHP4 were significantly lower in participants with low DHEA together with the glucocorticoids, cortisol, cortisone, CORT and 11DHCORT. Active glucocorticoids, cortisol and CORT, activate both the glucocorticoid receptor and the mineralocorticoid receptor (MR) with a binding affinity comparable to its natural ligand, ALDO. Regarding the glucocorticoids, 11βHSD2 has a regulatory function converting cortisol and CORT to cortisone and 11-DHCORT respectively. The enzyme thus prevents the activation of the MR by cortisol and CORT, both of which circulate at concentrations far exceeding those of ALDO. The lower glucocorticoid levels in the group with low DHEA were not due to deficient CYP11B since the precursor steroids of CORT and cortisol, 11-deoxycorticosterone and 11-deoxycortisol, respectively were not present in high concentrations. These two steroids were either too low to be detected or too low for accurate quantification. A further observation was that in participants with higher cortisol levels also had 11OHA4 concentrations higher than 5 nmol/L, highlighting the involvement of the adrenal enzyme, CYP11B1, in both glucocorticoid and adrenal androgen production.

Our findings show that in the participants with lower DHEA, fewer steroids were metabolised in adrenal pathways with altered steroids in the glucocorticoid and androgen pathways which speculatively, may be due to an impaired adrenal function possibly relating to signalling pathways involving corticotropin-releasing hormone and adrenocorticotropic hormone within the hypothalamic–pituitary–adrenal (HPA) axis. Furthermore, participants with low DHEA showed a significant decrease of cortisol:cortisone. Despite this, it is worth noting that ratios between 3.9 and 11 are considered normal; therefore males in the low DHEA group were still within the normal range, albeit at the lower end.

Our data emphasise the heterogeneity of steroid levels in healthy indivuals, highlighting the need to identify and account for this variability in the investigation of abnormal conditions. If one considers the participants as a single group, all participants appear to be within the norm. Analysis of a single group would not readily identify the participants with different profiles associated with lower circulating DHEA. In addition participants with altered adrenal steroid production in specific pathways would not be obvious in a single group analysis. Furthermore steroid concentrations measured in participants with lower DHEA would skew normal ranges of a single cohort. Confirmation of this finding and its underlying mechanism will require a much larger cohort and associated laboratory studies.

This study demonstrates that in health 11KT was the lowest of the 11-oxy androgens and ± 7.6-fold lower than 11OHA4. However the pattern changed in the participants with lower DHEA levels as 11KT is only 4.7-fold lower than 11OHA4 and 11OHT now the lowest of the C11-oxy androgens. This may be of particular importance in the pathogenesis and diagnosis of androgen related disease, for example, high levels of the 11-oxy androgens in PCa and BPH tissue are reported, particularly of 11KT, 11KDHT and 11KP4, with 11KT predominantly in the unconjugated form accumulating in prostate tissue^12,13^. 11KT is the predominant circulating active C11-oxy androgen in CRPC, suggesting it is a potential driver of CRPC via the AR^10^. In addition, 11-oxy androgen levels for 11OHA4, 11KT and 11KAST are associated with metastasis-free survival^25^. There is increasing interest in 11KT and its role in clinical androgen excess, with two studies reporting significantly increased 11KT in CAH patients compared to controls^6,26^.

The major strength of this study was the population of healthy men engaging in routine exercise with a narrow age range, medication free with accurate steroid quantification. The shortcomings of our analytical method can be overcome in future studies by focusing on specific steroids related to the investigation and including fewer steroids specific to the investigation. Altered methodology derivatising steroids may improve and enable the detection of steroids present at low concentrations. The finding of a subset of individuals within a cohort of normal young men with low circulating DHEA and altered adrenal steroid production is novel. This exploratory study will facilitate the powering of a definitive study to confirm these findings. Larger studies, covering wider weight categories and accounting for ethnicity, are needed to investigate the A4 and A5 level differences found. This may be particularly relevant for DHT that was not detected in some participants in the higher body weight range. Furthermore, the study highlights that in cases of androgen conditions related to either excess or deficiency, the measurement of T alone is not enough.

In conclusion, steroids other than testosterone significantly contribute to circulating bioactive androgens. We have identified, within a cohort of heathy participants, men with significantly lower DHEA and comparable T but altered adrenal steroids with lower circulating glucocorticoids and lower 11-oxy androgens. Findings highlight steroid heterogeneity in normal young men as well as the contribution of CYP11B1 to glucocorticoid and androgen production. Thus, this study holds importance in terms of the steroids that were identified, as well as those that went undetected, within this cohort of healthy men and may allow the better diagnosis and management of androgen related conditions.

## Materials and methods

### Reagents

Steroids were purchased from Sigma-Aldrich (St. Louis, USA), Steraloids (Newport, USA) and IsoSciences (Pennsylvania, USA) and deuterated steroid reference standards from Cayman Chemical company (Ann Arbor, Michigan, USA), CDN Isotopes (Augsburg, Bavaria, Germany) and Cambridge Isotopes (Andover, USA). Waters *UHPSFC-MS/MS* Quality Control (QC) reference material containing trans-stilbene oxide, thymine, sulfamethoxazole and sulfamethizole was purchased from Waters corporation (Milford, USA). Analytical-grade methanol, formic acid, and methyl *tert*-butyl ether (MTBE) were purchased from Sigma-Aldrich (St. Louis, USA).

### Study cohort

Healthy males, 71, between 18 and 35 years were eligible study inclusion and recruited by advert. Exclusion criteria: acute or chronic disease, acute or chronic abuse of alcohol, medication intake including counter drug preparations. Data collection took place between September and December 2018. Complete medical histories and medical examinations were undertaken. Fasting blood samples were collected between the hours of 08:00 and 09:00 am to circumvent diurnal variation, centrifuged and the serum was stored at -80 °C until analysis. Informed consent was obtained from all subjects/participants and the study was approved by the Institutional Review Board of Weill Cornell Medicine Qatar (Study 1284681-2, IRB no 18000012) and performed in accordance with the Declaration of Helsinki.

### UHPSCF-MS/MS analysis

Steroid levels were quantified by validated UHPSFC-MS/MS, allowing the separation of the C_19_ and 11-oxy C_19_ steroids, the C_21_ and 11-oxy C_21_ steroids, glucocorticoids and mineralocorticoids in a single chromatographic step^27^. Steroids are listed in Suppl. Table 1 indicating IUPAC and trivial names and abbreviations. Steroids, reference standards and quality control compounds were analysed in multiple reaction monitoring (MRM) mode using positive electrospray ionisation (ESI +) mode, with MS parameter settings together with quantifiers and qualifiers as previously reported. Response factors were calculated for steroid impurities in steroid standards and crosstalk interference between the labelled and unlabelled standards was avoided using unique MRMs. The limit of detection (LOD), lower limit of quantification (LLOQ), calibration range (ng/mL) and linearity (r^2^) for each steroid is indicated in Suppl. Table 2.

Free, unconjugated steroids were extracted from 0.5 mL thawed serum using a liquid–liquid extraction method. Samples were spiked with 50 µL internal standard mix containing 15 deuterated steroids (suppl. file), extracted with 5 mL MTBE (10:1 ratio) as reported, subsequent dried residues were resuspended in 150 µL 50% methanol for analysis. Masslynx 4.1 software (Waters Corporation) was used for data collection and processing.

### Statistical analysis

Data analysis was carried out using RStudio (version 4.2.2, RStudio, Boston, MA, US). Analyses were carried out to assess differences in participants with DHEA falling within the normal range (≥ 7 nmol/L) and those with low DHEA concentrations (< 7 nmol/L).

Descriptive statistics (means, standard deviations, medians, and interquartile ranges for continuous variables; counts and percentages for categorical variables) were used to summarise the demographic characteristics of these groups. Differences between groups were examined using Mann–Whitney tests for continuous demographic variables, while Fisher exact tests were used to examine differences in categorical variables. Mann–Whitney U tests also examined steroid levels between participants in the two groups.

For each steroid, the proportion of participants for whom the steroid was not detected (ND) below the LOD and that were detected exceeding the LOD and LLOQ, were calculated. Descriptive statistics, including means, standard deviations, medians, interquartile ranges, and ranges were reported for all steroids, regardless of whether they were detected or exceeded the LOD and LLOQ. The values of undetected steroids were held at zero 1/2 LOD. Differences in steroid levels between the normal and low DHEA groups were examined using Mann–Whitney tests. Finally, Chi-squared tests examined the association between the proportion of participants with DHEA levels above the LLOQ and the proportion of participants with steroid levels above the LLOQ for all other steroids. An a-priori alpha level of 0.05 was selected for all analyses.

### Ethics approval and consent to participate

All procedures performed in studies involving human participants were in accordance with the ethical standards of the Institutional Review Board of Weill Cornell Medicine Qatar (Study 1284681-2, IRB no 18000012) and performed in accordance with the Declaration of Helsinki and its later amendments or comparable ethical standards.

### Supplementary Information

Supplementary Tables.

## Data Availability

All the data for this study will be made available upon reasonable request to the corresponding author.
