# Supplementary material for: Circulating adrenal and gonadal steroid hormones heterogeneity in active young males and the contribution of 11-oxy androgens
Source: Sci Rep. 2024 Jul 14;14:16226. doi: 10.1038/s41598-024-66749-9 (PMC11246537; doi:10.1038/s41598-024-66749-9)
Supplement: Supplementary file 1 — Supplementary Tables. [file 41598_2024_66749_MOESM1_ESM.pdf]

Circulating adrenal and gonadal steroid hormones heterogeneity in active young males and the contribution of 11-oxy androgens

Amanda C. Swart[1,2#*] Desmaré van Rooyen[1], Therina du Toit[1], Bianca Heyns[1,2], John Molphy[3], Mathew Wilson[4], Roisin Leahy[5] and Stephen L. Atkin[6,7#]

[1] Department of Biochemistry,[2] Department of Chemistry and Polymer Science, Stellenbosch University, Stellenbosch 7600, South Africa

[3] Research Institute of Sport and Exercise Sciences, Liverpool John Moores University, Liverpool, United Kingdom

[4] Institute of Sport, Exercise and Health, University College London, London WC1E 6BT, United Kingdom

[5] Data Science Centre, School of Population Health, Royal College of Surgeons in Ireland, University of Medicine and Health Sciences, Dublin 2, Ireland

[6] Royal College of Surgeons in Ireland, Bahrain

[7] Weill Cornell Medicine Qatar, Doha, Qatar

Ultra high-performance supercritical fluid chromatography and tandem mass spectrometry.

The ACQUITY UHPSF chromatographic system was coupled to a Xevo TQ-S triple quadruple mass spectrometer (Waters Corporation, Milford, USA). Steroids (Suppl. Table 1) were separated on a Viridis® SFC ethylene-bridged hydrid (BEH) 2-ethylpyridine (2-EP) column (3.0 x 100 mm, 1.7 µm) fitted with a VanGuard™ pre-column (2.1 x 5 mm, 1.7 µm) (Waters corporation, Milford, USA). The following MS conditions were used: column temperature set to 60 °C, automated back pressure regulator to 1800 psi, capillary voltage of 3.8 kV, source temperature 120 °C, desolvation temperature and gas flow rate were set to 500 °C, and 1000 L/h, cone gas 150 L/h, nebuliser gas flow set to 7 bar and collision gas flow 0.15 mL/min. Post-column infusion of 1 % formic acid in methanol was pumped at a constant flow rate of 0.2 mL/min.

Supplemental Table 1. IUPAC names, trivial names and abbreviations of reference steroids included in the UPC^2-MS/MS analysis of circulating steroids of adrenal and gonadal origin.

IUPAC name	Trivial name	Abbreviation
**Adrenal origin**		
4-pregnen-11β,21-diol-3,18,20-trione	aldosterone	ALDO
4-pregnen-11β,18,21-triol-3,20-dione	18-hydroxycorticosterone	18OHCORT
4-pregnen-21-ol-3,11,20-trione	11-dehydrocorticosterone	11DHCORT
4-pregnen-11β,21-diol-3,20-dione	corticosterone	CORT
4-pregnen-21-ol,3,20-dione	deoxycorticosterone	DOC
4-pregnen-17α,21-diol-3,20-dione	deoxycortisol	
4-pregnene-11β,17α,21-triol-3,20-dione	cortisol	-
4-pregnen-17,21-diol-3,11,20-trione	cortisone	-
4-pregnen-11α-ol-3,20-dione	11α-hydroxyprogesterone	11αOHP4
4-pregnen-11β-ol-3,20-dione	11β-hydroxyprogesterone	11βOHP4
4-pregnen-3,11,20-trione	11-ketoprogesterone	11KP4
4-pregnen-11β,17-diol-3,20-dione	21-deoxycortisol	21dF
4-pregnen-17-ol-3,11,20-trione	21-deoxycortisone	21dE
5α-pregnan-11α-ol-3,20-dione	11α-hydroxydihydroprogesterone	11αOHDHP4
5α-pregnan-11β-ol-3,20-dione	11β-hydroxydihydroprogesterone	11βOHDHP4
5α-pregnan-3,11,20-trione	11-ketodihydroprogesterone	11KDHP4
5α-pregnan-11β,17α-diol-3,20-dione	11β-hydroxyPdione	11OHPdione
5α-pregnan-17-ol-3,11,20-trione	11-ketoPdione	11KPdione
5α-pregnan-3α,11β-diol-20-one	3,11dihydroxydihydroprogesterone	3,11diOHDHP4
5α-pregnan-3α-ol-11,20-dione	alfaxalone	
5α-pregnan-3α,11β,17α-triol-20-one	11β-hydroxyPdiol	11OHPdiol
5α-pregnan-3α,17α-diol-11,20-dione	11-ketoPdiol	11KPdiol
4-androsten-11β-ol-3,17-dione	11β-hydroxyandrostenedione	11OHA4
4-androsten-3,11,17-trione	11-ketoandrostenedione	11KA4
4-androsten-11β,17β-diol-3-one	11β-hydroxytestosterone	11OHT
4-androsten-17β-ol-3,11-dione	11-ketotestosterone	11KT
5α-androstan-11β-ol-3,17-dione	11β-hydroxyandrostanedione	11OH5αDIONE

5α-androstan-3,11,17-trione	11-ketoandrostanedione	11K5αDIONE
5α-androstan-11β,17β-diol-3,11-dione	11β-hydroxy-dihydrotestosterone	11OHDHT
5α-androstan-17β-ol-3,11-dione	11-ketodihydrotestosterone	11KDHT
5α-androstan-3α,11β,17β-triol	11β-hydroxyandrostanediol	11OH3αDIOL
5α-androstan-11-one-3α,17β-diol	11-ketoandrostanediol	11K3αDIOL
5α-androstan-3α,11β-diol-17-one	11β-hydroxyandrosterone	11OHAST
5α-androstan-3α-ol-11,17-dione	11-ketoandrosterone	11KAST
**Adrenal/Gonadal origin**		
5-pregnen-3β-ol-20-one	pregnenolone	P5
5-pregnen-3β,17α-diol-20-one	17α-hydroxypregnenolone	17OHP5
4-pregnen-3,20-dione	progesterone	P4
4-pregnen-17α-ol-3,20-dione	17α-hydroxyprogesterone	17OHP4
4-pregnen-16α-ol-3,20-dione	16α-hydroxyprogesterone	16OHP4
5α-pregnan-3,20-dione	dihydroprogesterone	DHP4
5α-pregnan-3β-ol-20-one	epi-allopregnanolone	epiAllo
5α-pregnan-17α-ol-3,20-dione	Pdione	Pdione
5α-pregnan-3α,17α-diol-20-one	Pdiol	Pdiol
5β-pregnan-3α,17,20α-triol	pregnanetriol	
5-androsten-3β-ol-17-one	dehydroepiandrosterone	DHEA
5-androsten-3β,17β-diol	androstenediol	A5
4-androstene-3,17-dione	androstenedione	A4
4-androsten-17β-ol-3-one	testosterone	T
5α-androstan-3,17-dione	androstanedione	5αDIONE
5α-androstan-17β-ol-3-one	dihydrotestosterone	DHT
5α-androstan-3α,17β-diol	androstanediol	3αDIOL
5α-androstan-3α-ol-17-one	androsterone	AST

Waters UPC^2 Quality Control (QC) reference standards ranging from 0.075 to 750 ng/mL were interspersed with samples during analysis to monitor chromatographic performance. A calibration curve (Supple. Table 2) was generated by plotting the ratio of the quantifier peak area and the internal standard

Supplemental Table 2. LOD, LLOQ, calibration range in ng/mL and linearity (r^2) of the calibration range of the steroids and QC samples. Reproduced with permission [28].

Steroid metabolite	LOD		LLOQ		Calibration range	r^2
	ng/mL	pg on column	ng/mL	pg on column		
5αDIONE	0.1	0.67	1	6.67	1 - 1000	0.9998
DHP4	0.2	1.33	1	6.67	0.2 - 1000	0.9999
P4	0.02	0.133	0.1	0.67	0.02 - 1000	0.9999
A4	0.2	1.33	1	6.67	1 - 1000	0.9994
11K-5αDIONE	1	6.67	2	13.33	1 - 1000	0.9974
11KDHP4	0.2	1.33	1	6.67	1 - 1000	0.9992
11KA4	0.2	1.33	1	6.67	0.2 - 1000	0.9999
AST	10	66.67	20	133.33	10 - 1000	0.9998
Pdione	20	133.33	100	666.67	20 - 1000	0.9999
11KP4	0.2	1.33	1	6.67	0.2 - 1000	0.9999
DOC	1	6.67	1	6.67	1 - 1000	0.9999
DHT	0.1	0.67	0.2	1.33	0.2 - 1000	0.9997
11OH5αDIONE	2	13.33	10	66.67	2 - 1000	0.9997
DHEA	2	13.33	10	66.67	2 - 1000	0.9998
Epiallopregnanolone	1	6.67	2	13.33	2 - 1000	0.9996
P5	2	13.33	10	66.67	10 - 1000	0.9996
11βOHDHP4	2	13.33	10	66.67	2 - 1000	0.9999
17OHP4	0.2	1.33	1	6.67	1 - 1000	0.9999
T	0.1	0.67	0.2	1.33	0.1 - 1000	0.9992
11KAST	1	6.67	2	13.33	1 - 1000	0.9998
11OHA4	0.2	1.33	1	6.67	1 - 1000	0.9998
11KPdione	2	13.33	10	66.67	2 - 1000	0.9998
11αOHDHP4	2	13.33	10	66.67	2 - 1000	0.9993
Alfaxalone	0.2	1.33	1	6.67	1 - 1000	0.9996
11KDHT	0.2	1.33	1	6.67	1 - 1000	0.9999
11βOHP4	0.2	1.33	1	6.67	0.2 - 1000	0.9986
11-DHCORT	0.2	1.33	1	6.67	1 - 1000	0.9999
16OHP4	0.02	0.133	0.1	0.67	0.02 - 1000	0.9999
21-dE	0.1	0.67	0.2	1.33	0.1 - 1000	0.9997
3αDIOL	10	66.67	20	133.33	10 - 1000	0.9992
11αOHP4	0.1	0.67	1	6.67	0.1 - 1000	0.9998
11OHAST	10	66.67	10	66.67	10 - 1000	0.9998
A5	2	13.33	10	66.67	10 - 1000	0.9993
Pdiol	1	6.67	2	13.33	1 - 1000	0.9997
11KT	0.2	1.33	1	6.67	1 - 1000	0.9998
deoxycortisol	0.2	1.33	1	6.67	0.2 - 1000	0.9998
11OHPdione	2	13.33	10	66.67	2 - 1000	0.9997
17OHP5	2	13.33	10	66.67	2 - 1000	0.9998
3α,11β-diOHDHP4	1	6.67	2	13.33	2 - 1000	0.9999
Corticosterone	0.2	1.33	1	6.67	0.2 - 1000	0.9998
11OHDHT	0.2	1.33	1	6.67	1 - 1000	0.9999
Pregnanetriol	1	6.67	2	13.33	1 - 1000	0.9995
Aldosterone	1	6.67	2	13.33	1 - 1000	0.9995
21-dF	0.2	1.33	1	6.67	1 - 1000	0.9998
cortisone	0.02	0.133	0.1	0.67	0.02 - 1000	0.9996

11K3αDIOL	0.2	1.33	1	6.67	0.2 - 1000	0.9994
11KPdiol	0.2	1.33	1	6.67	1 - 1000	0.9995
11OHT	0.02	0.133	0.1	0.67	0.02 - 1000	0.9997
Prednisone	0.2	1.33	1	6.67	0.2 - 1000	0.9981
11OHPdiol	1	6.67	2	13.33	2 - 1000	0.9998
11OH3αDIOL	10	66.67	20	133.33	10-1000	0.9995
cortisol	0.002	0.013	0.02	0.133	0.002 - 1000	0.9999
18OHcorticosterone	0.2	1.33	1	6.67	0.2 - 1000	0.9995
**QC compounds**						
(+/-) Trans-stilbene oxide	3.75	7.5	7.5	15	7.5-750	0.9992
Thymine	3.75	7.5	7.5	15	7.5-750	0.9997
Sulfamethoxazole	<0.075	<0.15	0.075	0.15	0.075-750	0.9972
Sulfamethizole	0.75	1.5	0.75	1.5	0.75-750	0.9997

peak area over the concentration using a linear regression equation and a $^1/_x$ weighting scheme. The internal standard mix with which all samples were spiked contained 15 deuterated steroids in absolute ethanol. (1 ng 4-pregnen-11β,17-diol-3,20-dione (2,2,4,6,6,21,21,21-D8) (21dF-d8), 1.5 ng 4-androsten-11β-ol-3,17-dione (2,2,4,6,6,16,16-D7) (11OHA4-d7), 5 ng 5α-pregnan-3,20-dione (1,2,4,5,6,7-D6) (DHP4-d6), 4-pregnen-11β,17,21-triol-3,20-dione (9,11,12,12-D4) (cortisol-d4), 5α-androstan-17β-ol-3-one (2,2,4,4-D4) (DHT-d4), 5α-androstan-17β-ol-3,11-dione (16,16,17A-D3) (11KDHT-d3), 4-androsten-17β-ol-3,11-dione (16,16,17A-D3) (11KT-d3), 10 ng 4-pregnen-3,20-dione (2,2,4,6,6,17A,21,21,21-D9) (P4-d9), 4-pregnen-17α-ol-3,20-dione (2,2,4,6,6,21,21,21-D8) (17OHP4-d8), 4-androsten-17β-ol-3-one (1,2-D2) (T-d2), 4-androsten-3,17-dione (2,2,4,6,6,16,16-D7) (A4-d7), 15 ng 5α-androstan-3α-ol-17-one (16,16-D2) (AST-d2), 20 ng 5α-androstan-3β-ol-11,17-dione (9,12,12,16,16-D5) (11keto-etiocholanolone-d5), 25 ng 5α-androstan-3α,17β-diol (16,16,17A-D3) (3αAdiol-d3), and 5-androsten-3β-ol-17-one (2,2,3,4,4,6-D6) (DHEA-d6).

Supplemental Table 3. Comparison of demographic characteristics participants with circulating DHEA lower than 7 nmol/L and DHEA above 7 nmol/L.

	DHEA >7 nmol/L (N=47)	DHEA <7nmol/L (N=24)	P-value
**Age**			
Mean (SD)	24.4 (4.66)	26.5 (4.75)	0.0664
Median (Q1, Q3)	23.0 (21.0, 27.0)	27.0 (22.8, 29.5)	
**Ethnicity**			
Arab	28 (59.6%)	10 (41.7%)	0.31
Black	10 (21.3%)	9 (37.5%)	
White	7 (14.9%)	5 (20.8%)	
Asian	2 (4.3%)	0 (0%)	
**Exercise**			
Mean (SD)	12.9 (4.63)	14.3 (7.18)	0.49
Median (Q1, Q3)	12.0 (12.0, 14.0)	13.0 (10.0, 18.0)	
**Height (cm)**			
Mean (SD)	180 (11.9)	184 (10.3)	0.188
Median (Q1, Q3)	178 (168, 187)	183 (176, 189)	
**Body Mass (kg)**			
Mean (SD)	76.6 (15.2)	82.5 (14.6)	0.0941
Median (Q1, Q3)	70.9 (65.1, 86.8)	83.7 (73.7, 91.6)	
**BMI**			
Mean (SD)	23.5 (2.40)	24.4 (3.23)	0.193
Median (Q1, Q3)	23.1 (21.4, 24.9)	24.4 (21.9, 25.8)	
**BMI Category**			
Underweight/normal	35 (74.5%)	13 (54.2%)	0.107
Overweight/obese	11 (23.4%)	10 (41.7%)	
**BSA**			
Mean (SD)	1.96 (0.251)	2.05 (0.219)	0.108
Median (Q1, Q3)	1.86 (1.74, 2.09)	2.05 (1.91, 2.23)	

Supplemental Table 4. Association between DHEA and other steroids**.**

	DHEA above LOD (n=55)	DHEA below LOD (n=15)	P-value
**A4**			
Above LLOQ	38 (70.4%)	10 (66.7%)	0.761
Below LLOQ/ND	16 (29.6%)	5 (33.3%)	
**11OHA4**			
Above LLOQ	53 (98.1%)	14 (93.3%)	0.390
Below LLOQ/ND	1 (1.9%)	1 (6.7%)	
**11KA4**			
Above LLOQ	1 (1.9%)	0 (0%)	1.000
Below LLOQ/ND	53 (98.1%)	15 (100%)	
**T**			
Above LLOQ	54 (100%)	15 (100%)	n/a
**11OHT**			
Above LLOQ	49 (90.7%)	12 (80.0%)	0.358
Below LLOQ/ND	5 (9.3%)	3 (20.0%)	
**11KT**			
Below LLOQ/ND	54 (100%)	15 (100%)	n/a
**DHT**			
Above LLOQ	13 (24.1%)	3 (20.0%)	1.000
Below LLOQ/ND	41 (75.9%)	12 (80.0%)	
**A5**			
Below LLOQ/ND	54 (100%)	15 (100%)	n/a
**P5**			
Below LLOQ/ND	54 (100%)	15 (100%)	n/a
**17OHP5**			
Above LLOQ	7 (13.0%)	0 (0%)	0.333
Below LLOQ/ND	47 (87.0%)	15 (100%)	
**P4**			
Above LLOQ	7 (13.0%)	0 (0%)	0.333
Below LLOQ/ND	47 (87.0%)	15 (100%)	
**16OHP4**			
Above LLOQ	42 (77.8%)	4 (26.7%)	<0.001
Below LLOQ/ND	12 (22.2%)	11 (73.3%)	
**17OHP4**			
Above LLOQ	0 (0%)	1 (6.7%)	0.217
Below LLOQ/ND	54 (100%)	14 (93.3%)	
**CORT**			
Above LLOQ	54 (100%)	14 (93.3%)	0.217
Below LLOQ/ND	0 (0%)	1 (6.7%)	
**cortisone**			

	DHEA above LOD (n=55)	DHEA below LOD (n=15)	P-value
Above LLOQ	54 (100%)	15 (100%)	n/a
**cortisol**			
Above LLOQ	54 (100%)	15 (100%)	n/a
**11-DHCORT**			
Above LLOQ	54 (100%)	14 (93.3%)	0.217
Below LLOQ/ND	0 (0%)	1 (6.7%)	
